# Olfactory Event-Related Potentials and Exhaled Organic Volatile Compounds: The Slow Link Between Olfactory Perception and Breath Metabolic Response. A Pilot Study on Phenylethyl Alcohol and Vaseline Oil

**DOI:** 10.3390/brainsci9040084

**Published:** 2019-04-15

**Authors:** Sara Invitto, Andrea Mazzatenta

**Affiliations:** 1 Department of Biological and Environmental Science and Technologies, University of Salento, Campus Ecotekne, Via per Monteroni, 73100 Lecce, Italy; 2 DReAM Laboratory of InterDisciplinary Research Applied to Medicine, University of Salento-Vito Fazzi Hospital, 73100 Lecce, Italy; 3 Dipartimento di Neuroscienze, Imaging e Scienze Cliniche, Università “d’Annunzio” di Chieti-Pescara, 66100 Chieti, Italy; amazzatenta@unite.it

**Keywords:** OERP, VOCs, N1, LPC, olfactory perception, olfactory metabolic response

## Abstract

Olfactory processing starts with the breath and elicits neuronal, metabolic and cortical responses. This process can be investigated centrally via the Olfactory Event-Related Potentials (OERPs) and peripherally via exhaled Volatile Organic Compounds (VOCs). Despite this, the relationship between OERPs (i.e., N1 and Late Positive Component LPC) and exhaled VOCs has not been investigated enough. The aim of this research is to study OERPs and VOCs connection to two different stimuli: phenylethyl alcohol (PEA) and Vaseline Oil (VO). Fifteen healthy subjects performed a perceptual olfactory task with PEA as a smell target stimulus and VO as a neutral stimulus. The results suggest that OERPs and VOCs distributions follow the same amplitude trend and that PEA is highly arousing in both psychophysiological measures. PEA shows ampler and faster N1, a component related to the sensorial aspect of the stimulus. The N1 topographic localization is different between PEA and VO: PEA stimulus evokes greater N1 in the left centroparietal site. LPC, a component elicited by the perceptual characteristic of the stimulus, shows faster latency in the Frontal lobe and decreased amplitude in the Central and Parietal lobe elicited by the PEA smell. Moreover, the delayed time between the onset of N1-LPC and the onset of VOCs seems to be about 3 s. This delay could be identified as the internal metabolic time in which the odorous stimulus, once perceived at the cortical level, is metabolized and subsequently exhaled. Furthermore, the VO stimulus does not allocate the attentive, perceptive and metabolic resource as with PEA.

## 1. Introduction

### 1.1. Olfactory Function and Breath

The olfactory function, placed in a cortical localization connected to areas dedicated to emotional and mnestic activations (e.g., hippocampus, entorhinal cortex, amygdala), seems to be a borderline function between “perceptions”. It plays a role linked to instinctual emotional responses, evolutionarily less recent both from the phylogenetic and the ontogenetic point of view [1,2]. Moreover, the olfactive function strongly involves and modulates metabolic aspects [3,4], and this peculiarity is more clearly overt if compared to other sensory modalities. Different to olfactory animal models [5,6,7,8], the olfactory whole pathway, linked to human perceptions, has been largely unexplored and needs full investigation because it is a complex sense that has not only a sensorial/perceptive finality but guides internal chemical cues, such as metabolism [3]. Humans can perceive the presence of an odorant according to its concentration (perceptual threshold) [9], with the concentration depending on the single odorant chemical molecule taken into consideration [10,11]. Many brain structures detect olfactory connections. There is an “olfactory map” based on inhibitory and excitatory interactions within and between the two olfactory bulbs [12]. The response of neurons in the bulbs is also subject to modulation through a top down regulation. The precise identification of an odour requires further processing after the physiological stages of the olfactory system. The axons afferent from the olfactory bulb form the olfactory tract and branch out to reach different regions of the frontal lobe, including the olfactory cortex. The neocortex is only reached by some branches from the median dorsal nucleus of the thalamus. It is important to recall that the organization of the olfactory system is unique, because the olfactory system does not have decussation and does not relay to the thalamus [13,14]. This could indicate its extremely ancient origin at the phylogenetic level and could be linked to the more basic physiological aspects (e.g., metabolic/oxidative aspects) [4,15,16]. Furthermore, the sense of smell is mainly modulated by a breath-dependent sensory gate [16,17,18].

### 1.2. Chemosensory Event Related Potentials 

Chemosensory Event Related Potentials (CSERP) and the Olfactory Event Related Potentials (OERP) are the main psychophysiological and electroencephalographic tools used to study the olfactory responses due to chemical stimulus (e.g., odorants) [19]. The registration of CSERP was clinically introduced by Kobal in the early 1970s [20]. CSERPs have become appropriate tools to evaluate the olfactory function, the olfactive sensation and perception [21,22,23]. The characteristic components of CSERPs are N1 and Late Positive Component (LPC). N1 is an early component with negative polarity, reflects the sensory characteristics of the stimulus and depends on the exogenous component. N1 and LPC seem to vary in amplitude and latency in relation to the concentration of odorants (i.e., higher odorant concentration can elicit shorter latency in N1 and ampler amplitude in LPC) [23]. Moreover, in contrast to the other sensory modalities, the effects of selective attention on odours lead to a reduction of the latency instead of an increase of the amplitude in the N1 component.

The LPC is a slow positive component, which follows the N1 and which reflects the perceptive and cognitive characteristics of the stimulus itself and depends on the endogenous components [24]. 

The LPC component has, instead, the probable function connected to the perceptual-cognitive information processing. We can consider the LPC, evoked by the olfactory stimulation, as a delayed P3 in a cognitive task.

Moreover, it has been shown that aging is accompanied by a decrease in OERP sensitivity, as well as a greater tendency to olfactory adaptation and a slower recovery of threshold sensitivity [20,25]. In any case, the analysis of the olfactory cortical responses must consider some specific methodological biases. The olfactory stimulation usually occurs in a mono-lateralized way (in a single nostril) but this modality can produce cortical lateralization bias, due to both tactile stimulation and odorous stimulation itself. Additionally, some responses to odorous stimuli can activate the trigeminal system, rather than the olfactory system. For example, stimuli such as ammonia, acids or carbon dioxide, activate the trigeminal system and subcortical components linked to nociception [26,27]. As with other sensory modalities, the sense of smell is also susceptible to changes in aging [27], in different cognitive capacity [28], depend on stimulus and is task-related [29,30]; these variations are viewable through the EEG. 

### 1.3. The Volatile Organic Compounds (VOCs)

In a human of healthy condition, 99% of the exhaled breath matrix is composed of a few compounds of the inorganic gasses such us nitrogen, oxygen and carbon dioxide along with vapor aqueous and the inert gases. The residual part consists of a mixture of many molecules: un-volatile organic compounds (e.g., leukotrienes, prostaglandins and serotonin) and by volatile organic compounds (e.g., aldehydes, ketones and benzene derivatives) [31,32]. The VOCs are divided into exogenous and endogenous: the exogenous VOCs are derived from the environment or human habits (e.g., smoker), while the endogenous VOCs are the result of the body’s metabolic processes (e.g., glucose degradation originates acetone; cholesterol biochemical pathway of the mevalonic acid derives isoprene; fat acids peroxidation produces alkanes). Gas exchange at the alveolar-blood capillary membrane of the respiratory tract is a passive diffusion driven by concentration gradients. Following these vital gasses, molecules present in the blood can diffuse passively into the breath. In normal subjects, more than 3400 different VOCs can be detected in the exhaled breath [32]. The VOCs’ profile in exhaled breath reflects the biochemical alterations related to metabolic changes, organ failure, or neuronal dysfunction in disease, which are, at least in part, transmitted via the lung to the alveolar exhaled breath, even at the very onset of disease [32,33,34,35]. In recent studies, VOCs analysis has been applied to neurodegenerative diseases [36,37,38] and cognition [33]. Interestingly, VOC evaluation in a healthy centenarian shows a peculiar pattern in comparison with young and elderly controls [35], as was the case for OERP [27,39,40]. Neuronal metabolism in some diseases induce VOC alteration, as suggested by these studies, and moreover there is a lack of studies on physiological neuronal activity and VOCs release.

### 1.4. Connection between OERP and VOC

The connection between olfactive perception and metabolic response is not well investigated. Mazzatenta et al. focused on metabolic aspects (i.e., VOCs) and oxidative processes in some neurodegenerative pathologies [34,38,41]. Invitto’s recent studies investigated the OERP variation in Mild Cognitive Impairment [28] and in Obstructive Sleep Apnoea (OSA) syndrome [16], both pathologies that show altered VOCs [34,38]. This aspect is particularly evident in OSA, where the altered breath compromises metabolic and oxidative capacity [16,42,43]. It is certainly known that the respiratory act is the gateway necessary for the activation of the olfactory receptor response [44], but we know nothing about the metabolic activity produced as a function of different stimuli and how this activation is correlated with the sensory response. In the same way, we know that breath can interact with ERP amplitude and EEG rhythms in different ways [45,46,47], but we do not know enough about a parallel investigation into VOCs and OERPs elicited by the same condition (e.g., same stimulus, same cognitive task or same attentional condition). 

In the present study, we investigated the trend between OERP and the exhaled VOCs during the PEA sensorial (rose/floral smell) stimulation and the Vaseline Oil (neuter smell) sensorial stimulation [48], focusing in particular on N1 and LPC OERPs components, the main components elicited during an olfactory perceptive task [16,28].

## 2. Materials and Methods

### 2.1. Participants

We recruited 15 healthy adult volunteers (10 females; mean age 24.4; standard deviation 4.4). The subjects were university students. All the subjects were non-smokers. The subjects had normal hearing, normal or corrected-to-normal vision and a right manual dominance. All participants signed a written informed consent according to the Helsinki Declaration. The protocol was approved by the local ethical committee (Ethical Committee ‘Vito Fazzi Hospital’ ASL Lecce, Italy—authorization with report number 36-2016) 

### 2.2. OERP Apparatus and OERP Processing

In the present task, we administered olfactory stimuli via an olfactometer [49] interfaced in parallel with the EEG amplifier. This tool allows the administration of scented stimuli triggered on line on EEG recordings [16,28,49,50]. Continuous EEG was recorded (sampled at 500 Hz) during the olfactory task, via the V-AMP-Brain Vision Recorder, using a cap embedded with 16 Ag /AgCl electrodes (Brain Products, Germany), positioned according to the 10–20 system electrode. Impedance was kept below 5 kΩ. Electrodes were referenced online to the FCz [51], and offline referenced as common averaged reference [52]. One electrode was placed at the outer canthus of the left eyebrow and was used to monitor eye movements. The ERPs analysis was obtained using the Brain Vision Analyzer and the time off-line analysis was from 100 pre-stimulus to 600 ms post-stimulus with 100 ms baseline-correction. Thereafter, trials with blinks and eye movements were rejected based on horizontal electro-oculogram with an ICA component analysis. The signal was filtered offline (0.01–50 Hz, 24 dB), and the threshold for artefact rejection was set at > |125|μV [16,28]. Ocular rejection was performed through independent component analysis (ICA). Separate averages were calculated for each odorant segmentation (PEA and Vaseline Oil). Peaks detection was applied according to the latency range of the maximum peak area in Grand Average [16,28].

### 2.3. VOC Apparatus 

During the EEG recordings subjects were, in parallel, recorded with the iAQ-2000 (Applied Sensor, Warren, NJ, USA) equipped with a metal oxide semiconductor (MOS). The e nose has a sensing range of 450–2000 ppm CO_2_ equivalents and is able to detect a broad range of volatile compounds (both organic and inorganic, e.g., alcohols, aldehydes, aliphatic hydrocarbons, amines, aromatic hydrocarbons, ketones, organic acids, and CO), while correlating directly with the CO_2_ levels [32,33,34,35].

### 2.4. Method

The subject was sitting comfortably in the laboratory setting, where there was a constant temperature of 20 degrees centigrade. The subject was sitting in front of a black box (interfaced with the olfactometer) and his task was to try to have an orthonasal breathing and to perceive the odorous stimuli during the task.

The sequence of stimulus presentation S1 (500 ms) and S2 (for 500 ms) was administrated in a pseudorandom way, with a proportion of 50% of the target (i.e., PEA smell). The interstimulus interval was 1 min during which the individual was exposed only to air. The air flow, after collecting the volatile components of substances S1 and S2, was transferred to an exposure open black box, where the subject was required to enter his head. In this paradigm the stimulation was administrated in the central way and in a diffused manner (not in only one nostril), to avoid lateralization bias. The outlet of the tube from which the essences emerged was placed at the centre of the stimulation cave, where the subject had to inhale and exhale. Each experimental session had a duration of about 40 min. 

After the Task, the experimenter asked the subject if he had perceived the smell and if he perceived the odour, and if the odour was pleasant, arousing or familiar on a scale from 1 to 5 (i.e., 1 = minimum value; 5 = maximum value) [53].

In order to record OERPs correlated to physiological effect on VOCs emission, we interfaced the olfactometer to the EEG and to an electronic sensor for VOCs parallel recording. This condition allowed us to control olfactory stimulations and to relate them to psychophysiological responses. Consequently, we detected: the olfactory response to record, for each given single step of stimulation, the EEG, the OERPs components and VOCs responses. The experimental settings allowed us to investigate, through OERPs and VOCs, the olfactory response to a neutral stimulus (Vaseline Oil 10 mL added of 20 μL) and to a scented stimulus of a characteristic rose odour (PEA, 2-phenyl ethanol C_2_H_4_O_2_ with a dilution of 20 μL in 10 mL). 

## 3. Statistical Analysis and Results

### 3.1. Statistical Analysis

VOCs data normalization was made by 1/log10. Data treatment and statistical analysis was performed with Excel, Origin and SPSS software, α was set at 0.05. OERP data analysis was performed with SPSS Software

#### 3.1.1. OERP Analysis 

The General Linear Model (GLM), repeated measure, was performed on amplitudes and latencies of N1 and LPC component; as the Within Factor we considered Smell (PEA, Vaseline Oil), and Electrodes (Fp1; Fp2; F3; F4; F7; F8; C3; C4; P7; P8; O1; O2; Fz; Cz; Pz)

#### 3.1.2. N1

The analysis on N1 latency showed significant results on Electrode (*F* =2.42; *p* = 0.004; η^2^ = 0.157) and on Smell × Electrode (*F* = 2.15; *p* = 0.011; η^2^ = 0.142). Post hoc *t* Test paired comparison highlighted significant differences in terms of an N1 faster latency elicited by PEA stimulation in the Frontal Right localization (F4 PEA smell = 207 ms; vs. F4Neuter Smell = 246.6 ms).

**Figure 1:**
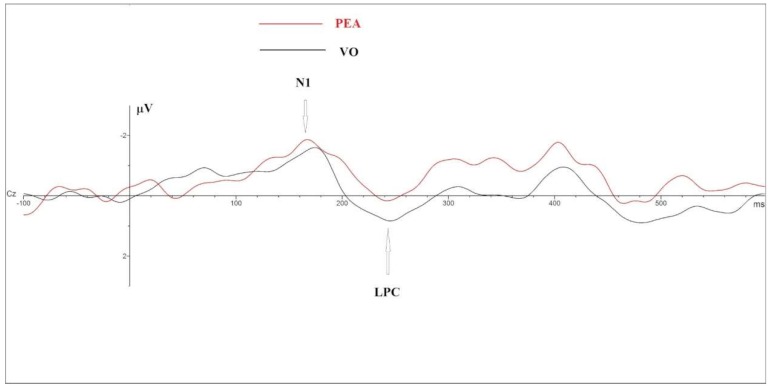
Cz–Grand Average comparison between OERP components elicited during phenylethyl alcohol (PEA) (red line) and Vaseline Oil (black line).

**Figure 2:**
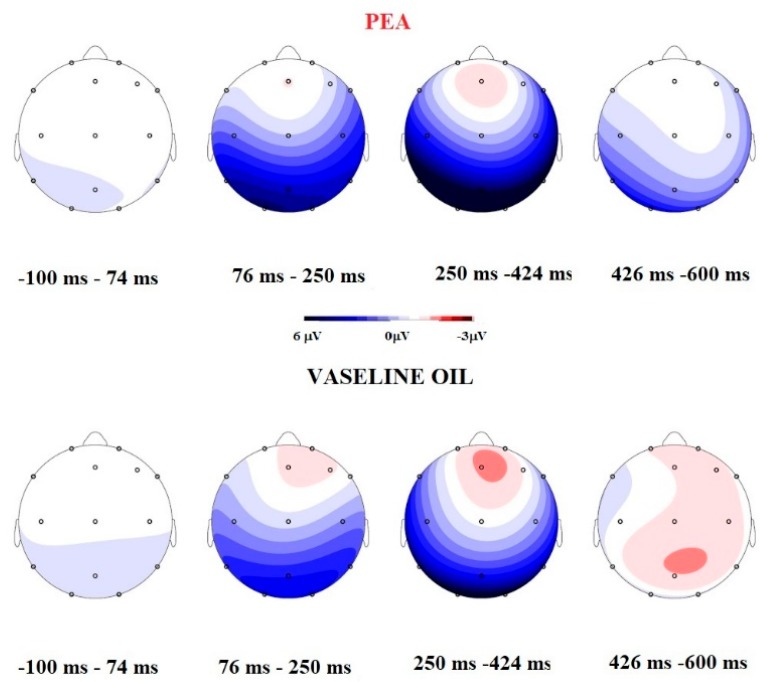
Comparison between Mapping during four different temporal ranges and between OERP component. The Mapping during PEA stimulation indicates that is present a more negative activation in the temporal range 250–425 ms, and positive components during the Vaseline Oil stimulation. The OERP amplitude comparison shows that PEA elicited ampler N1 and decreased late positive component (LPC).

GLM on N1 amplitude highlighted the main significant effect on Smell (*F* =31.45; *p* = 0.000; η^2^ = 0.724) and Electrode (*F* = 19.9; *p* = 0.000; η^2^ = 0.624) and an interaction on Smell × Electrode (*F* = 4.674; Sign = 0.000; η^2^ = 0.280). PEA stimulation elicited ampler N1 (PEA = −7.5 µV vs. VO = −5.4 µV); post hoc *t* Test paired comparison showed significant difference in F4, C3, C4, P7; P8, O1, O2, F8, Cz, Pz in term of ampler N1 in PEA stimulation (see Table 1 and Figure 1 and Figure 2).

#### 3.1.3. LPC

The GLM on LPC latency showed a significant effect only in Electrode variable (*F* = 2.450; *p* = 0.003; η^2^ = 0.159) in term of shorter latencies in Frontal topography (see Table 2).

GLM on LPC Amplitude showed a significant Interaction between Smell × Electrode (*F* = 2.075; *p* = 0.015; η^2^ =0.147). Post Hoc paired *t* Test comparison highlighted significant results in Cz (*t* = 2.44; *p* = 0.030; VO mean amplitude = 2.702 µV; PEA mean amplitude = 2.095 µV) and in Pz (*t* = 2.356; *p* = 0.035; VO mean amplitude = 6.54 µV; PEA mean amplitude = 4.97 µV), in terms of a decreased amplitude elicited by PEA stimulation (see Figure 1). 

**Figure 3:**
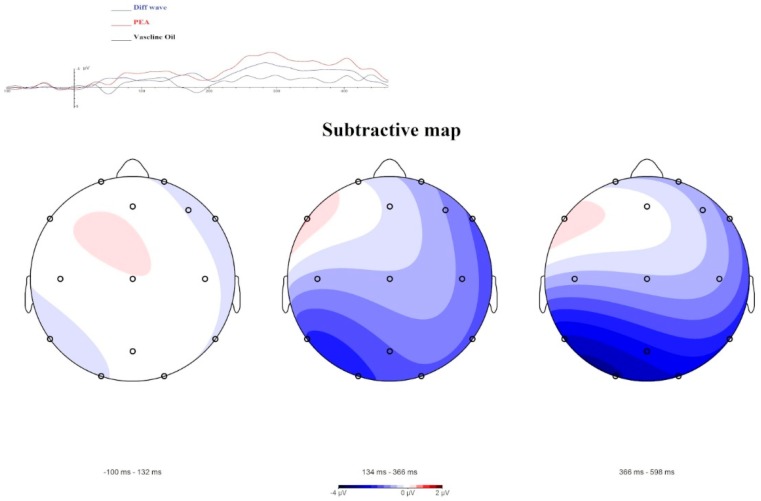
A subtractive visualization of the OERP component and of Mapping. The blue line of OERP component shows the difference wave between PEA condition and Vaseline Oil condition. The Mapping representation highlights a negative activity (linked to N1 results) shifted in a delayed temporal range (306–600 ms). This negative amplitude is related to the odorant characteristic of the stimulus.

Moreover, the OERP components and Maps obtained (Figure 3), through data comparison (i.e., difference waves between PEA administration and Vaseline Oil administration), highlight greater negativity with a left lateralization.

### 3.2. VOCs Analysis 

**Figure 4:**
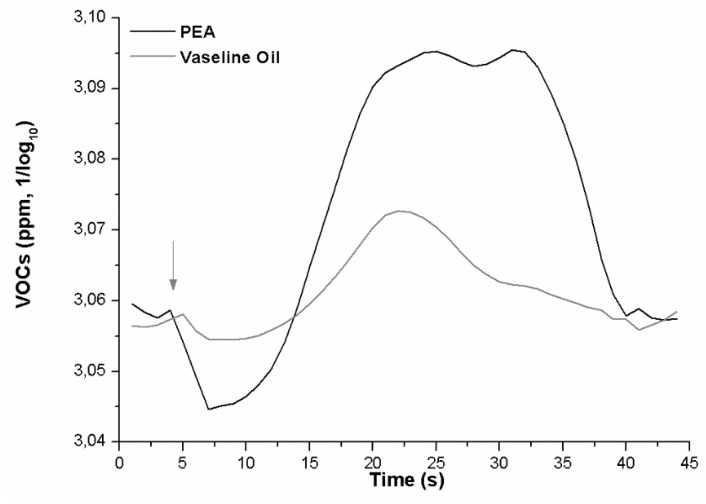
Grand average curves of the volatile organic compounds (VOCs) response to PEA (black) and Vaseline oil (grey). The exhaled VOCs metabolic response is about 8 s delayed, by the stimulus administration (arrow), and the recovery occurs after about 20 s.

A paired t-test comparison was performed on VOCs Amplitude and Latency. The test returned a significant difference between the VOCs exhaled after PEA administration versus control (*t* = 5.466; *p* = 0.012; PEA mean amplitude = 3.08; standard deviation = 0.02; VO mean amplitude = 3.06; standard deviation= 0.006) (Figure 4) in term of greater VOC amplitude elicited by PEA smell.

The analysis of latency highlighted that the onset of the VOCs variation showed dissimilar values (*t* = 2.917; *p* = 0.019; PEA onset = 3.56 ms; VO onset = 6.89 ms). 

### 3.3. Behavioral Results

One hundred percent of the subjects reported they perceived the PEA smell during the task. The perceived smell was judged with a low score for the pleasantness (mean = 1.31; standard deviation = 1.01); with low scores for familiarity (mean = 1.8; standard deviation = 1.12) and with a low score for arousal (mean = 1.61; standard deviation = 1.4).

## 4. Discussion and Conclusions

### 4.1. OERPs and VOCs can Be Comparable

Recent literature highlights how respiration and metabolic activity can affect ERP components [45,46,47,54] and in particular how the olfactory system is strongly connected to metabolic and respiratory activity [4,41]. However, we are not aware of how our cortical system is related to respiratory metabolic activity. Being in the early stages of this research topic, it seemed particularly useful to work on a single sensorial/anatomic channel that is linked to the olfactory system. Therefore, our investigation is focused on how the olfactory system produces, through the receptor and cortical response, a perceptual variation induced by stimulation and how and when the metabolic system could be affected. Another open question is whether the distributions of the evoked answers (OERP and VOCs) are somehow comparable/superimposable. VOCs and OERPs analysis, through an electrophysiological and psychophysiological approach, can be an interesting starting point to begin an investigation of this evaluation. 

The research data show that, in our setting, the N1—a component closely related to the sensory/perceptive characteristics of stimulation [21]—is more sensitive to highlighting differences between the two different stimuli. In particular, our results highlight a significant difference between PEA and Vaseline oil, in the direction of an ampler and faster N1 elicited by PEA stimulation and a decreased LPC in Cz and Pz electrode site. This point can indicate that subjects perceive the PEA smell at a sensorial level and have an allocation of perceptive resource for Vaseline Oil, where the sensorial connotation of the smell is absent. Furthermore, the sites of N1 cortical localization for PEA and Vaseline Oil are different: PEA stimulus evokes greater N1 in the left centroparietal lobe, while Vaseline Oil evokes N1 in an equivalent way, even if less ample, both in the right and left hemisphere. Subtractive Maps indicate that the odour perception (obtained by subtracting the activation acquired with PEA from that with Vaseline Oil), is localized in the left centrotemporal lobe. At the behavioural level the subjects evaluated the PEA odour as having little activating effect, as unfamiliar and unpleasant. The exhaled VOCs show a curve with a trend that starts with a downward deflection and then grows towards increased values. The exhaled VOCs’ amplitudes follow the trend of the OERP results. The results suggest that VOCs are highly sensitive to stimuli and that the PEA stimulus is highly discernible compared to the control. Moreover, the maximum amplitude visible in the grand average of the VOCs is present for around 20 s and thereafter there is a refractory time in which the sensor continues to record the VOCs present in the air, but in decay.

### 4.2. The Slow Link between Olfactory Perception and VOC Metabolic Response

According to our data, this temporal range present from OERP (a temporal range between baseline time 0 and 600 ms) to VOCs (a temporal range between 600 ms and about 3.5 s) could be the internal metabolic time for which the odorous stimulus, once perceived at the cortical level, is metabolized and subsequently exhaled. In the expiration phase therefore, volatile chemical compounds have their concentration time in the area and decay (temporal range between 2.7 and 40 s). This level of concentration is equivalent to the level of cortical processing of the stimulus. The lateralization showed in the left hemisphere for PEA smell could be considered in this case an attempt to understand and discriminate the stimulation at the conscious level, as the stimulus PEA can be categorized as a floral stimulus or as the smell of a rose. On the other hand, current OERP and VOCs data suggest that the Vaseline Oil stimulus does not allocate the attentive, perceptive and metabolic resource as the PEA does. At the most integrated level, this psychophysiological bridge between cortical activation and metabolic response is a slow pathway because it requires, among other things, the involvement of involuntary and voluntary motor acts, such as respiratory ones. 

### 4.3. Limitation of the Study and Pathway for Future Research

Our future goal will be to investigate, in OERP and VOCs, what happens in the temporal range from a time of 0 to 3 s at the sensory, metabolic and perceptive levels, after sensorial and perpetual olfactive stimulation. Moreover, we aim to evaluate this connection through a survey of the exhaled VOCs analysed through their chemical components and the connection between several different odorants. We think that the perception of a pleasant or unpleasant or neutral substance can prompt different metabolic mechanisms to action and therefore produce different endogenous cascade activations. 

A significant limitation of this work included not being able to analyse the VOCs in their main components and in their chemical definitions, elements which therefore did not allow us to thoroughly investigate how central processes can guide peripheral processes but has allowed us exclusively to observe times and overlaps of characteristics of components. In any case, this work is the basis, albeit limited, for developing future research full of possibilities. 

## Figures and Tables

**Table 1:** Table 1 describes significant results of Post Hoc paired *t* Test comparison: *t* Value, *p*, N1 mean amplitude (in µV) of PEA and VO.

Electrode	*t* Value	*p*	PEA	VO
F4	3.664	0.003	−3.9	−2.4
C3	3.173	0.007	−6.19	−4.48
C4	3.062	0.009	−6.82	−4.89
P7	4.429	0.001	−12.25	−9.06
P8	5.040	0.000	−13.21	−9.23
O1	4.865	0.013	−14.17	−11.26
O2	3.957	0.002	−14.95	−11.15
F8	2.67	0.019	−8.60	−5.54
Cz	2.724	0.017	−3.17	−2.49
Pz	4.332	0.001	−10.31	−8.06

**Table 2:** Table 2 points out the descriptive values of Latencies (in ms) for each electrode.

Electrode	Latency	SEM
Fp1	389.73	23.7
Fp2	358.13	28.36
F3	348.67	26.18
F4	367.63	27.55
C3	413.6	26.42
C4	386	31.06
P7	416	28.5
P8	404.4	30.74
O1	424.53	25.69
O2	424.40	24.5
F7	384.93	25.5
F8	348.53	34.05
Cz	426.53	23.19
Pz	447.07	18.95
Fz	338.27	20.82
